# MOMP, cell suicide as a BCL-2 family business

**DOI:** 10.1038/cdd.2017.179

**Published:** 2017-10-20

**Authors:** Halime Kalkavan, Douglas R Green

**Affiliations:** 1 Department of Immunology, St. Jude Children’s Research Hospital, 262 Danny Thomas Place, Memphis, TN 38105, USA

## Abstract

Apoptosis shapes development and differentiation, has a key role in tissue homeostasis, and is deregulated in cancer. In most cases, successful apoptosis is triggered by mitochondrial outer membrane permeabilization (MOMP), which defines the mitochondrial or intrinsic pathway and ultimately leads to caspase activation and protein substrate cleavage. The mitochondrial apoptotic pathway centered on MOMP is controlled by an intricate network of events that determine the balance of the cell fate choice between survival and death. Here we will review how MOMP proceeds and how the main effectors cytochrome *c*, a heme protein that has a crucial role in respiration, and second mitochondria-derived activator of caspase (SMAC), as well as other intermembrane space proteins, orchestrate caspase activation. Moreover, we discuss recent insights on the interplay of the upstream coordinators and initiators of MOMP, the BCL-2 family. This review highlights how our increasing knowledge on the regulation of critical checkpoints of apoptosis integrates with understanding of cancer development and has begun to translate into therapeutic clinical benefit.


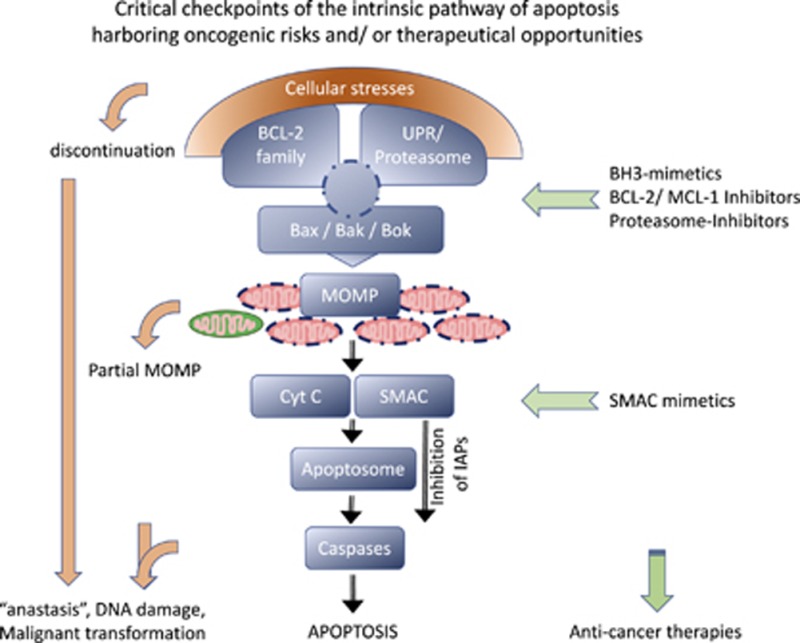


Graphical Abstract

## Facts

- The interaction of BCL-2 family members orchestrates mitochondrial outer membrane permeabilization (MOMP) and can thereby translate cellular stresses into apoptosis initiation.
- In addition to protein–protein interactions within the BCL-2 family, BCL-2 effector proteins can be regulated directly by post-translational modifications.
- The mitochondrial intermembrane space harbors proteins (e.g., cytochrome *c*, SMAC, Omi) that are involved in caspase activation.
- Apoptosis via the intrinsic pathway requires nearly complete MOMP; partial MOMP can lead to pathophysiology.

## Open Questions

- How do specific BCL-2 family members relate to cell-type-dependent stress responses?
- How does BOK (BCL-2-related ovarian killer) integrate into a model of the mitochondrial pathway of apotosis?
- How do mitochondrial membrane proteins impact on the release of cytochrome *c*?
- How do mitochondrial dynamics and metabolism impact MOMP?

Since the evolution of multicellular species, the decision of a cell to live or to die in response to a potential threat to its functional coherence has become important not only for the individual cell itself but also for the cell’s community – the tissues, organs, and organism. Ongoing research into the biology of cell death uncovered effector mechanisms that enable us to classify different cell death pathways. Broadly, these are often divided into three major types of cell death: apoptosis, autophagy, and necrosis,^1, 2, 3, 4^ although the second remains poorly understood (and often misinterpreted). The fact that cell death can take place in a programmed and ordered way, as in apoptosis and regulated necrosis (e.g., necroptosis), provides a potential opportunity to manipulate the cell fate choice between death or survival. However, the exploitation of cell death pathways for long-term therapeutic benefit requires an in-depth understanding of the molecular mechanisms underpinning cell death and their (patho)physiological relevance.

**Figure 1:**
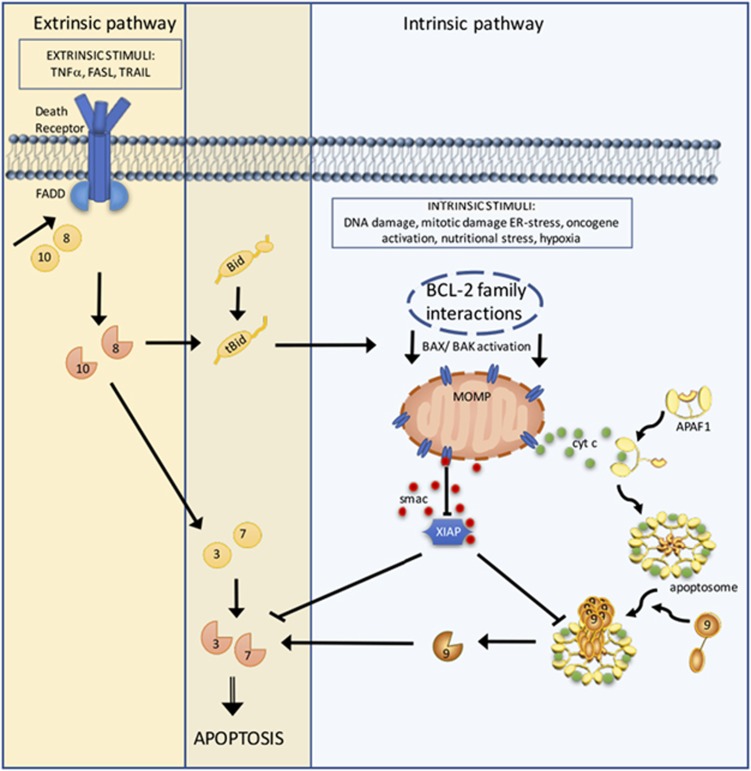
The extrinsic and intrinsic apoptotic pathways. The extrinsic pathway is initiated by engagement of DRs via their respective ligands TNF, FASL/CD95L, or TRAIL. Together with the adaptor FAS-associated death-domain (FADD) protein and the initiator procaspase-8 (or -10) they form the death-inducing signaling complex (DISC). This assembly enables the dimerization and autoactivation of the initiator caspases, which in turn cleave and activate the executioner caspase-3 and -7, ultimately leading to apoptosis unless they are inhibited by XIAP. The intrinsic pathway can be engaged by diverse intracellular stresses that modulate BCL-2 family protein interactions that control the activation of the BCL-2 effector proteins BAX and BAK. Once activated, BAX and BAK cause MOMP, leading to the release of proapoptotic IMS proteins. Cytochrome *c* (Cyt *c*) engages APAF1 and induces its oligomerization, leading to apoptosome formation that recruits and activates the initiator procaspase-9. Active caspase-9 cleaves and activates the executioner caspase-3 and -7. Simultaneously with Cyt *c*, Smac is released from the IMS and inhibits XIAP. The extrinsic and intrinsic pathways are linked; caspase-8 can cleave the BH3-only protein BH3-interacting domain death agonist (Bid), leading to its active, truncated form tBid, which in turn activates BAX/BAK. Numbers in circles indicate the respective pro- and active caspase; interrupted circles represent active caspases

Apoptosis occurs predominantly via two main pathways, often referred to as the extrinsic and the intrinsic pathways, depending on the apoptosis-initiating signal (Figure 1).^5^ The extrinsic pathway is initiated via death receptors (DRs) at the cell membrane. The intrinsic (mitochondrial) pathway, on the other hand, is controlled by the BCL-2 protein family and is executed by mitochondrial outer membrane permeabilization (MOMP). Both pathways lead to the activation of cysteine proteases, specific for aspartic acid residues, that is, caspases.^6^ Ultimately, caspase-3 and -7, the executioners of apoptosis, cleave more than 1000 different proteins leading to their degradation or activation. This in turn affects physiological processes including chromatin 'remodeling', manipulation of signaling pathways, deformation of the cytoskeleton, and exposure of signals for phagocytosis and clearance of the dying cell.^7, 8^ As a result of this multitude of enzymatic reactions, apoptotic cells display a typical phenotype characterized by chromatin condensation (pyknosis), cell shrinkage, and membrane blebbing.^9^

Predominant in vertebrate cells is the intrinsic pathway of apoptosis in which diverse forms of cell stressors can modulate the composition and/or activation status of pro- and antiapoptotic Bcl-2 family members. These in turn engage MOMP, generally via the BCL-2 effector proteins BCL-2-associated X protein (BAX) and BCL-2 antagonist or killer (BAK). MOMP results in the release of proapoptotic intermembrane space (IMS) proteins that promote the formation of a molecular complex called the apoptosome, leading to caspase-9 engagement and caspase-3 and -7 activation.^2^

The extrinsic pathway can be activated by ligand binding of a subset of the TNF receptor superfamily, including TNFR1, TRAIL receptors (DR4 and DR5) and Fas (Apo-1; CD95).^3^ This leads to the activation of caspase-8 and, as in the intrinsic pathway, ultimately to the engagement of caspase-3 and -7. Cells that are susceptible to apoptosis via direct activation of the caspase cascade by the extrinsic pathway, such as T lymphocytes, are designated type 1 cells. However, often the extrinsic pathway independent of MOMP is not sufficient to trigger cell death; a crosstalk between the extrinsic and intrinsic pathways exists that can amplify the extrinsic signal.^10^ For this purpose, caspase-8 cleaves the proapoptotic Bcl-2 protein BID to tBID (truncated), which then activates BAX and BAK.^11^ Cells that require this interaction for extrinsic apoptosis are referred to as type 2 cells (e.g., hepatocytes, pancreatic cells).

Characterization of the crucial players and interactions of the intrinsic pathway of apoptosis is of ongoing intense research effort, aiming to target the apoptosis machinery for successful clinical translation. Most recently, highly valuable mechanistical insights into BCL-2 family interactions enabled the development of cancer therapies, including the *bona fide* BH3-mimetic drugs (Navitoclax), a highly selective BCL-2 inhibitor (Venetoclax), and MCL-1 inhibitors.^12, 13, 14^ In addition to their therapeutical use, BH3 profiling of tumors has also been proposed as a 'biomarker' strategy for the prediction of chemotherapy sensitivity.^15, 16, 17^ Dissecting the mechanisms underlying apoptosis will allow us to exploit programmed cell death for the treatment of human diseases.

Here we review the central player of apoptosis, the mitochondrion, before highlighting the most recent insights into the Bcl-2 effector molecules causing MOMP. Finally, we discuss a unified theory of the role of the Bcl-2 family and their interactions focusing on how they implement the decision for the cell to live or die.

## MOMP, Releasing the Beasts

The most important functional properties of the mitochondria appear to be carried out on their membranes. The mitochondrial inner membrane (IMM) carries the respiratory chain that provides a source of energy via oxidative phosphorylation. Together with the IMM, the integrity of the mitochondrial outer membrane (OMM) is crucial to form a compartment – the IMS – harboring proteins that can induce and promote cell death if released to the cytosol. As such, cytochrome *c* and second mitochondria-derived activator of caspase (SMAC) embody the most crucial and best-studied IMS proteins involved in apoptosis.

The OMM is physiologically permeable to molecules up to 5 kDa.^18^ During MOMP pores are formed that allow proteins larger than 100 kDa to pass into the cytosol.^19^ Research so far is consistent with a model of MOMP in which the formation of pores in the OMM is achieved by the integration and conformational change of activated BAX and BAK. Per mitochondrion this process has a duration of seconds, but since the onset of MOMP for each mitochondrion in a cell varies, it usually requires ~5 min for all mitochondria within a cell to permeabilize.^20, 21^ In some instances, high-resolution imaging techniques allowed the observation of a wave-like propagation of MOMP within single cells.^22, 23^ Although mechanistically still unresolved, an argument exists that wave propagation might be executed via ER calcium channels.^23, 24^ However, the link to BCL-2 family proteins remains unclear, and elevation of intracellular calcium is not generally required for MOMP or apoptosis.

**Figure 2:**
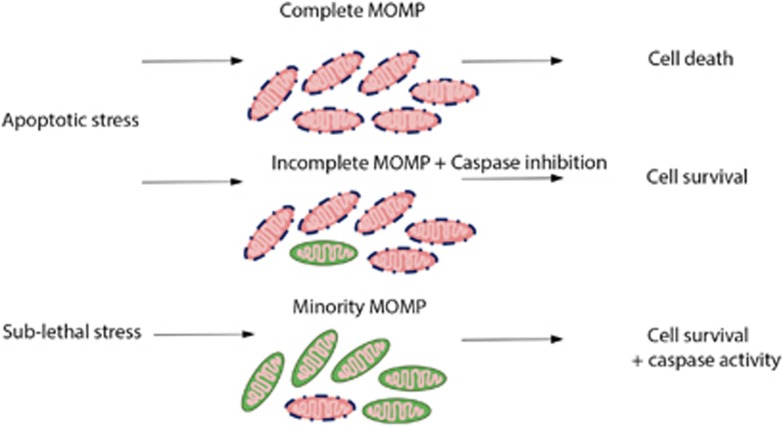
Overview of MOMP and its exceptional nuances. In general, apoptotic stress results in complete MOMP and apoptotic cell death. Scenarios of partial MOMP: iMOMP, in which case cells might survive if caspase activity is inhibited and minority MOMP, when only a small portion of mitochondria undergo MOMP. The latter version of partial MOMP results in cell survival, but caspase-dependent signaling pathways can be activated and DNA damage might occur, which might lead to malignant transformation of cells

Generally, MOMP has been considered to be an 'all or nothing' decision within a celI, as a mitochondrial consensus that leads inevitably to a cell’s death. As for any dogma, under specific circumstances the binary character of MOMP has been challenged. Intracellular heterogeneity in the engagement of MOMP has been reported that impacts on cell survival as well as differentiation.^10, 25, 26, 27^ To date, two different scenarios of partial MOMP that do not lead to cell death have been described: incomplete MOMP (iMOMP), meaning that MOMP occurred in most but not all mitochondria within a cell; and minority MOMP (we propose, for an abbreviation, 'miniMOMP'), in which only a few mitochondria experience MOMP after a sublethal stress (Figure 2). Survival of a cell that encounters apoptotic stress and iMOMP depends on the absence or inhibition of caspase activity.^28, 29, 30^ It has been proposed that iMOMP is the result of unequal antiapoptotic BCL-2 protein expression on mitochondria that can expand and repopulate the cell, supporting cell survival upon receipt of diverse apoptotic stimuli, provided caspase activity is inhibited.^28^ In contrast, miniMOMP induces caspase activity, but at sublethal levels.^31^ The consequences include DNA damage and other non-apoptotic caspase signaling functions, that can promote oncogenic transformation of a cell.^10, 31, 32, 33^ These observations of partial MOMP may enable a cell to engage a more nuanced response to proapoptotic events, and also underline the crucial function for an ultimate 'all or nothing' decision for the cell when it comes to MOMP and whether to proceed to apoptosis to avoid further damage. Similarly to MOMP, evidence exists that caspase activation itself can be nuanced or even reversible, if the death-inducing stress has been removed. It has been proposed to name this phenomenon anastasis ('rising to life').^34, 35^ Interestingly, the authors point out the evolutionary advantage of these cells surviving DNA damage, namely stress-induced genetic diversity.^36^

Taken together, although it was long thought that apoptosis engaged by tumor suppressors or by anticancer therapy ends survival of oncogenic transformed cells,^37^ failure of its completion may in fact promote cancer initiation and progression. Depletion of caspases in cancer cells has been described,^38^ and might enable cancer cells to survive diverse apoptotic stimuli (stresses, therapy) if the threshold for initiation of a complete MOMP is not reached. Moreover, the risk for secondary malignancies such as chemotherapy-associated leukemia^39, 40^ or irradiation-associated sarcomas^41^ might be increased via the induction of minority MOMP or in response to cellular stresses including some anticancer therapies.

## IMS Proteins and Caspase Activation

Upon MOMP, the IMS proteins, cytochrome *c* and SMAC (also known as DIABLO) together with other proteins, are released to the cytosol with similar kinetics independent of their size.^42^ Although evidence exists for a spatiotemporally coordinated release of IMS proteins, it remains controversial as to whether or not different IMS proteins can be released independently.

### Cytochrome *c*

Cytochrome *c* has a crucial role in respiration and in apoptosis. Although its intracellular location in healthy cells is within the mitochondrion, or more precisely in the IMS, it is encoded in the nucleus and first translated as apocytochrome *c*. From the cytosol it is translocated through the OMM to the IMS, where it is converted to the mature protein holocytochrome *c* by covalent linkage with the heme group via its synthetase.^43^ Mostly free in the IMS, cytochrome *c* has a crucial function in the electron transport of the respiratory chain between complexes III and IV. The remaining fraction of cytochrome *c* is tightly membrane bound to the IMM in healthy cells. The actual amount of IMM-bound cytochrome *c* is not well defined, but some evidence suggests that it accounts for more than 15% of the total.^43^ The anionic phospholipid cardiolipin (CL) was identified as the binding partner for the highly basic cytochrome *c*, mainly via electrostatic and hydrophobic bonds.^44, 45^ Moreover, this binding *per se* was associated with functional properties of cytochrome *c* as a (hydro)peroxidase, with CL itself as a substrate.^46, 47^ Structural characterization of cytochrome *c* and ultrafast x-ray spectrometry analysis revealed that a disruption of the heme–Met80 interaction within cytochrome *c* leads to a conversion from a hexa- to a pentacoordinate form with a free heme ligand.^48, 49^ Interestingly, it was shown that the hydrogen bonding network, and the entatic ('tensioned') state of cytochrome *c* needed for its function as an electron shuttle, is disrupted upon binding to lipids and related hydrocarbons, leading to the loss of the Met80 ligand and active-site conformational change, which in turn enabled peroxidase activity.^49, 50^ It remains unclear if the interaction between CL and cytochrome *c* leading to the oxygenation of CL and the proapoptotic peroxidase activity of cytochrome *c* has a pioneering role in the release of IMS proteins during MOMP.^47, 51, 52^

Evidence also exists for another protein that impacts on the release of cytochrome *c* from the IMS to the cytosol, the dynamin-related protein 1 (DRP1). DRP1 is known for its significant role in mitochondrial fission. In the context of MOMP, it has been shown that cells deficient for DRP1 have a significant delay in cytochrome *c* release, but the release of other IMS proteins may be unaffected.^53, 54, 55^

Although mechanistic insights into the release of cytochrome *c* are pending, its function in the cytosol as the initiator of the apoptosome is well established in vertebrates (Figure 1). Notably, *Caenorhabditis elegans* and *Drosophila* do not have this role of cytochrome *c* in apoptosis.^56^ On release into the cytosol, heme ligand carrying cytochrome *c* molecules bind monomers of apoptotic protease-activating factor 1 (APAF1) and cause their oligomerization into a heptameric wheel-like signaling platform in a dATP-dependent manner.^6, 19^ This assembly leads to a conformational change of APAF1 that reveals its N-terminal caspase recruitment domains (CARD). Known as the apoptosome, this sepatameric protein complex is now able to bind and activate procaspase-9.^57, 58^ Once activated, the initiator caspase-9 cleaves and thereby activates the executioner caspase-3 and -7, ultimately leading to apoptosis.^59^

### Second mitochondria-derived activator of caspase

SMAC is released to the cytosol almost simultaneously with cytochrome *c* upon MOMP, where it neutralizes the X-linked inhibitor of apoptosis protein (XIAP) and other IAPs (Figure 1).^42, 60^ XIAP is a cytosolic protein that binds and inhibits active caspase-3 and -7 and thereby can hinder apoptosis. The inhibitory effects of SMAC on IAPs are mediated by binding of its N-terminal AVPI motif to the BIR2 and BIR3 domains (baculoviral IAP repeat domains) of IAPs.^60, 61, 62^ The IMM protease PARL (presenilin-associated rhomboid like) is responsible for the activation of SMAC by generation of the amino-terminal IAP-binding motif.^63^ Therapeutic SMAC mimetics exploit the proapoptotic property of SMAC by binding to XIAP, cIAP1, and cIAP2 to enable apoptosis, or in the absence of caspase-8 activity, engage necroptosis.^4, 64^ The clinical translation of SMAC mimetics, for example, LCL161 or Birinapant, indicate a possible therapeutic benefit for ovarian cancer, lymphoma, and multiple myeloma.^65, 66^ However, further randomized clinical evaluation and combination therapies are outstanding and may reveal the clinical impact of SMAC mimetics in cancer therapy. There remains, however, an urgent need for biomarkers for the preselection of susceptible patients.^67^

Similar to SMAC, OMI/HtrA2 (48 kDa) is involved in the neutralization of IAPs.^68, 69, 70^ However, loss of either alone or both does not hinder apoptosis, suggesting that other IAP antagonists in the IMS are likely to function redundantly.^19^ Other IMS proteins such as endonuclease G or apoptosis-inducing factor are released during MOMP, but are mainly associated with caspase-independent cell death (CICD), although their requirements for CICD remain controversial.^71^

The role of MOMP in the extrinsic pathway of apoptosis in type 2 cells (see above) is frequently misunderstood as a need for cytochrome *c* release, APAF1, and caspase-9 function for apoptosis to occur. However, antiapoptotic BCL-2 proteins inhibit extrinsic apoptosis even in cells lacking APAF1.^72^ It is now clear that upon MOMP, the disinhibition of XIAP and its effects on executioner caspases, activated by caspase-8 in the extrinsic pathway, is responsible for this effect (Figure 1). This is best exemplified by *in vivo* experiments in which animals lacking BID are protected from lethal liver damage induced by ligation of the DR, CD95, while animals lacking both BID and XIAP are sensitive.^11^

## BCL-2 Family Effectors: How to Permeabilize the OMM

### BAX and BAK

BAX and BAK are indisputable BCL-2 effector proteins and MOMP executioners, and cells lacking both proteins are often unable to engage MOMP.^73, 74^ The subcellular localization of both proteins is crucial for their roles as MOMP executioners: in healthy cells, BAX is mainly located in the cytosol and the main fraction of BAK appears to be constitutively membrane bound on mitochondria. To trigger MOMP, BAX translocates to the OMM and BAK must be disengaged from antiapoptotic BCL-2 proteins such as MCL-1 and BCL-xL.^75, 76^ Intense research has been directed toward understanding the minimal requirement for BAX and BAK activation. Most evidence suggests a model that requires for their activation at least a transient interaction of the hydrophobic groove of BAX or BAK with BH3-only proteins at the OMM.^37, 77, 78, 79, 80, 81^ Well established from diverse experimental settings, authors named this transient interaction 'kiss-and-run'.^82, 83, 84^ The resulting conformational change in the BCL-2 effector proteins was considered necessary for dimer formation.^85, 86, 87^ In turn, these primary homodimers were deemed as 'the minimal unit' for the assembly of higher-order oligomers that effect MOMP.^84, 88, 89^

**Figure 3:**
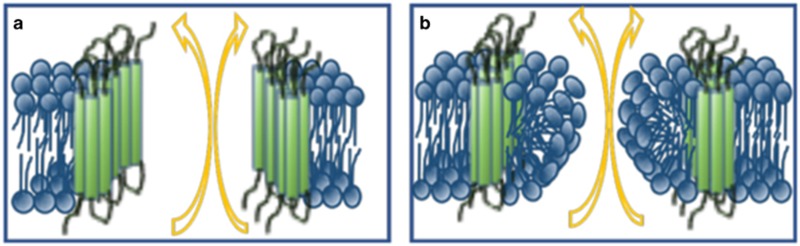
Schematic view of proposed models for structure of membrane pores. (**a**) Proteinaceous pores consist of BAX/BAK oligomers at the inner rim. (**b**) Lipid pores are also initiated and stabilized by BAX/BAK oligomerization, but the pore’s edge is formed by a monolayer of lipids, allowing hydrophilic proteins to pass the pore

However, understanding how BAX and BAK form pores in the OMM is the subject of extensive ongoing research. The two main theories regarding the pores responsible for permeabilization are centered on whether they are proteinaceous or lipidic (Figure 3). Proteinaceous pores comprise oligomers of BAX and/or BAK that form stepwise growing channels.^19, 90^ Super-resolution data visualized the rings formed by BAX oligomers in the OMM.^53^ However, this does not exclude a lipidic pore, taking the technical limitations into account. Other models of proteinaceous channels for MOMP are based on an expansion of preexisting channels, such as voltage-dependent anion channels (VDAC). However, although it was shown that BCL-2 effector proteins can interact with VDAC, deficiency of VDAC isoforms failed to show any impact on the intrinsic pathway of apoptosis.^57^

By contrast, increasing evidence points towards a lipidic nature of BAX/BAK-induced pores (also named toroidal or proteolipidic pores).^91^ The underlying biophysical explanation is that an increased membrane tension occurs when proteins are integrated asymmetrically into the lipid bilayer, which in turn leads to membrane instability and the opening of pores in the membrane. As the pore rim itself has a line of tension that creates a force for pore closure, thereby counteracting the membrane tension, the role for BAX and BAK in this toroidal model is mainly their integration into the pore edges, thereby decreasing the line tension and stabilizing the pore.^92, 93^ The (proteo)lipid pore model is most compatible with large, growing pores and is consistent with the technical difficulties in visualization of the pores.

### BCL-2-related ovarian killer

BCL-2-related ovarian killer (BOK, BCL2L9) is the most conserved BCL-2 family member.^94, 95^ Structurally it shares 70–80% sequence homology with BAX and BAK and is considered another potential BCL-2 family effector.^96^ Although first discovered in murine ovaries, BOK is present not only in reproductive tissues but also the brain, kidney, spleen and the gastrointestinal tract.^94, 97, 98^ Based on overexpression experiments in murine embryonic fibroblasts (MEFs), BOK was predominantly found to be membrane bound to the golgi and the endoplasmic reticulum (ER).^99^

Despite years of fruitful research on BOK, its role as a BCL-2 effector molecule remains unclear. Concerns about a functional role in apoptosis mainly arose from the observation that BOK-deficient mice generated independently in different laboratories by targeting distinctive exons^97, 98, 100^ have no overt phenotype. Taking the role of apoptosis in development and differentiation into account, which is defective in BAX and BAK double-deficient mice, the absence of abnormalities in BOK-null mice suggested that any crucial function for BOK as an effector protein for MOMP may not be related to development.^98^ However, it is likely that BAX, BAK, and BOK have redundant roles, and no published studies have yet explored the impact of BOK ablation in the BAX and BAK double-deficient background.

It also became obvious that the role and regulation of BOK in apoptosis must be distinct from BAX and BAK. Although possibly ontogenetically dispensable, BOK turned out to be crucial for ER-associated stress responses in some cells. However, *in vivo* evidence is still limited.^97, 100, 101^ Evidence exists that BOK is able to directly cause MOMP independently of BAX and BAK, but this proapoptotic activity is inhibited by the actions of the cytosolic arm of the ER-associated degradation (ERAD) pathway.^97, 102^ In these studies, cell lines expressing 'unstable' BOK sustain survival by active degradation of this MOMP effector.^97, 102^ Thus, the viable cell lines (e.g., MEF, HCT116) examined to date show very low or barely detectable protein levels of BOK,^99^ unless ERAD is disrupted.^97^ Overexpression or stabilization of BOK in BAX and BAK double-knockout MEF, either via proteasome inhibition with MG132 or with the valosin-containing protein (VCP) inhibitor ESI resulted in MOMP and apoptosis.^97, 102^

Interestingly, some studies indicate that BOK is often associated with inositol 1,4,5-trisphosphate receptors in the ER, which may hinder its proteasomal degradation. However, cytoplasmic 'free' BOK is considered to lead ultimately to the death of a cell.^99, 103, 104^

While increasing evidence suggests that BOK is a genuine, noncanonical MOMP effector independent of BAK and BAX, unresolved mechanistic and (patho)physiological questions remain. Fernandez-Marrero *et al.*^105^ provided evidence that BOK lacking the transmembrane C terminus (BOK^ΔC^) is able to form pores in artificial membranes of liposomes that mimic the composition of mitochondria. Moreover, the membrane permeabilization capacity of BOK^ΔC^ was enhanced by cleaved BID, but was not inhibited by BCL-XL. In contrast, in their experimental settings, BAX- and BAK-deficient mitochondria were resistant to BOK-induced MOMP. In other studies, no effects of BH3-only proteins or the antiapoptotic proteins, BCL-2, BCL-xL, or MCL-1, on BOK-mediated liposome permeabilization or apoptosis in cells was observed.^97^ Taking all findings so far into account, BOK might embody a BCL-2 effector protein able to perform MOMP independently of BAX and BAK that is distinctive in its function and regulation.

### The active role of the OMM

The active role of the OMM in the activation and inhibition of BAX and BAK has been the subject of intense and controversial research. Most recently, O’Neill *et al.*^106^ provided evidence that argues against the need for direct activation of BAX and BAK by other proteins, and supports a role for the OMM alone for the activation of the BCL-2 effectors. In their experiments, ablation of all known BH3-only proteins, as well as p53 and Rb, via CRISPR/cas9 technology, did not prevent the induction of MOMP in HCT116 colon carcinoma cells. In that setting, deletion of the C-terminal end (helix 9) was the only way to hinder membrane insertion of BAX or BAK and consequent MOMP induction.^57^ Although their approach makes a convincing argument, one may consider the possibility of yet unknown players that might confound the interpretation of the results, or a putative role for BOK as a potential BH3-only protein-independent effector in these cells.

In support of a role for the OMM in the activation of BAX and BAK, products of a sphingomyelin pathway were shown to interact with these BCL-2 effector proteins.^107^ ER-derived sphingomyelinase converts sphingomyelin to ceramide, and from there, to sphingosine at the OMM, which in turn is converted to sphingosine-1-phosphate (S1P) and the fatty aldehyde, hexadecanal. S1P binds to BAK, while hexadecanal binds to BAX. While low concentrations of hexadecanal facilitates the activation of BAX by active BID, higher concentrations appeared to activate BAX alone. Another study showed that TNF*α*-induced generation of ceramide at the mitochondria is sufficient to induce Bax translocation to mitochondria and subsequent MOMP.^108^ Therefore, it remains possible that products of this lipid pathway function in the activation of BAX and BAK, even in the absence of BH3-only proteins.

Other than its role in BAX and BAK activation, the OMM also has a critical role in the interaction of active BAX and BAK with prosurvival BCL-2 proteins.^109, 110, 111^ Known as the 'retrotranslocation' theory, it has been shown that cytosolic BAX and BAK spontaneously attach to the OMM, which promotes their homodimerization and MOMP, unless prosurvival members are present to heterodimerize with them at the OMM and cause their retrotranslocation into the cytosol.^112, 113^ Thus, it is thought that in healthy cells BAX and BAK are retained in the cytosol or continuously retrotranslocated by prosurvival proteins to avoid MOMP.^114^ Interestingly, the interaction and heterodimerization between proapoptotic BCL-2 family members and effectors also requires the presence of membranes.^115^ Additionally, recent research suggests an autoinhibitory mechanism of BAX by assembly of asymmetric, cytosolic homodimers.^116^

## The Unified Theory of BCL-2 Family Function, and Beyond

Understanding how the BCL-2 family integrate diverse intracellular signals into a cell fate decision between cell death or survival is a key issue and has been the focus of considerable effort. Thirty members of the BCL-2 family have been identified to date. Their structural homologies and functional properties enable us to classify the BCL-2 family proteins into three groups: the active effectors of MOMP (BAX, BAK, BOK) as discussed above, and the BH3-only proteins and antiapoptotic (prosurvival) proteins (e.g., BCL-2, BCL-x_L_, BCL-W, MCL-1, A1). Further, the BH3-only proteins are separated into direct activators (active BID, BIM, PUMA), which activate the effector proteins, and sensitizers or derepressors (e.g., BAD, NOXA), which neutralize the antiapoptotic proteins.^73^

**Figure 4:**
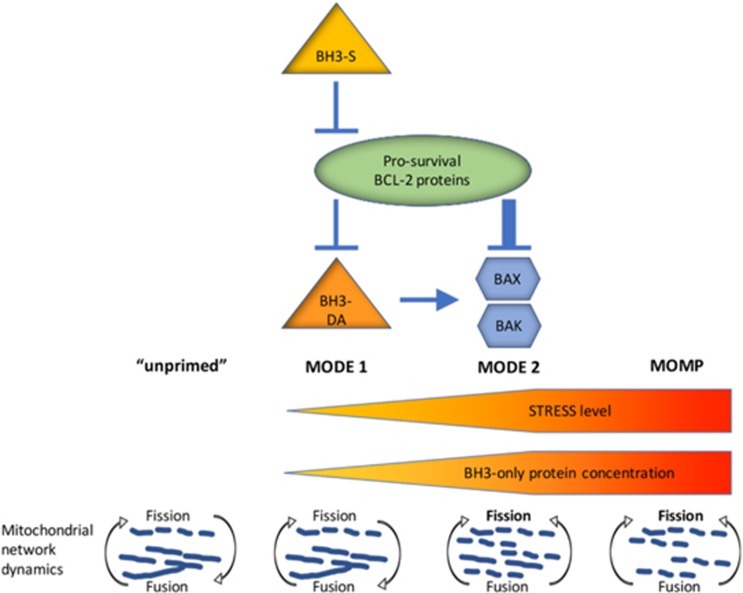
The unified model of BCL-2 protein interactions. In the 'unprimed', healthy cell, BAX and BAK reside in an inactive state that does not require active suppression of apoptosis via prosurvival BCL-2 family members. Cells 'primed for death' undergo MOMP with different kinetics upon derepression. In Mode 1, prosurvival proteins sequester BH3-only direct activators (BH3-DA). This reversible interaction can be easily overcome by BH3-only sensitizer (BH3-S) proteins. More effective inhibition of MOMP onset takes place in Mode 2, when prosurvival proteins directly sequester BAX and BAK under high-stress levels. Ultimately, when both modes of survival are overcome, BAX/BAK oligomerization leads to MOMP. Taking mitochondrial dynamics into account, in healthy cells inactive effectors BAX/ BAK support mitochondrial dynamics by promoting mitochondrial fusion. Increased activation of BAX/BAK leads to an imbalance in mitochondrial dynamics towards fission, leading to mitochondrial fragmentation

Several models have been proposed to simplify the complex network between the BCL-2 family members and induction of MOMP upon diverse cellular stresses.^73, 117^ They are known as the 'direct activation model',^118^ 'displacement model' (also named 'neutralization model'),^119^ 'embedded together model',^120^ and the 'unified model'.^110^ These diverse models have engendered some incoherence in published data based on different experimental models and different interpretations of the data available. The similarities and differences between the models and how they evolved have been reviewed extensively elsewhere^73^ and are not covered in detail here. Instead, we focus on the most recently proposed unified model (Figure 4).^110^

In healthy cells, BAX and BAK are proposed to be inactive and do not need to be controlled by antiapoptotic BCL-2 proteins.^110^ Cellular stresses lead to the activation of only a fraction of the effectors BAX and BAK by the direct activators, which in turn engages a feedforward autoactivation of the remaining pool of MOMP effectors.^110, 121^ Based on the embedded together model,^120^ antiapoptotic proteins are at the center of MOMP prevention upon cellular stresses. They can both sequester proapoptotic BH3-only proteins (Mode 1) and/or sequester BAX and BAK (Mode 2). Unlike the embedded together model, in the unified model the two modes are unilateral and Mode 2 is more efficient than Mode 1. Thus, the effect of sensitizer BH3 proteins on antiapoptotic BCL-2 family proteins has more impact on Mode 1 than on Mode 2. This difference is almost negligible under low-stress conditions when minor amounts of unbound direct activators lead to insufficient activation of BAX and BAK. However, high-stress conditions engage an increase of unbound direct activators capable of BAX and BAK activation. In this scenario, the more efficient Mode 2 comes into play and antiapoptotic proteins sequester BAX and BAK, resulting in delayed MOMP induction.

Consistent with the embedded together model, the unified model accredits a critical role for the OMM in the inhibition of active BAX and BAK by antiapoptotic BCL-2 proteins.^109, 110^ Further, and in contrast with former models, the unified model links a role for MOMP with mitochondrial dynamics; in Mode 2 oligomerized or sequestered BAX and BAK prevent mitochondrial fusion, resulting in fragmentation of the mitochondrial network.

The unified model is an attempt to predict how the apoptotic machineries are engaged upon different stress levels. However, it raises no claim for completeness, and further research is needed to differentiate not only the stress intensity but stress type as well as cell-type-dependent differences that define the regulatory dependence on a particular death or survival mode. Moreover, recent research suggests that there is an additional mode (Mode 3) of cell survival, which is independent of the expression of antiapoptotic BCL-2 members and their interactions, but rather relies on the degradation of the effector protein BOK.^97, 102^ No model of MOMP regulation so far integrates the potential role of BOK. However, ongoing research underlines a significant role for BOK under certain cellular stresses (e.g., ER stress), which needs to be incorporated into future models. Different from Modes 1 and 2, this regulatory mode appears to be independent of antiapoptotic BCL-2 family members and depends on the cytosolic arm of the ERAD following post-translational modifications (PTM). Similar roles of PTMs for BAX or BAK were observed, for example, BAX*β*, a ubiquitylated, constitutively active form of BAX. Yet, in contrast to BOK, modified BAX and BAK can still be antagonized by antiapoptotic proteins.^122^

## Concluding Remarks

The progress in the field of the intrinsic pathway of apoptosis is remarkable. Considerable innovations and improvements in imaging technologies and experimental design, including artificial membranes, and the contribution of structural biologists, have enabled in-depth – and often visual – investigations. However, partially inconsistent data must be resolved, and mechanistic insights remain elusive at multiple levels upstream, during, and downstream of MOMP. The processing of different cellular stresses, the dependence on cell type, and on preexisting constellations of BCL-2 family members within each cell are still to be defined. MOMP, as a bilateral process between the cell, represented by BCL-2 effector proteins, and the mitochondria, represented by IMS- and membrane-bound proteins, requires further characterized. Increasing evidence points toward a crucial role of the (proteo)lipids of the OMM and IMM, but their interactions with BAX, BAK and BOK as well as with IMS proteins deserves more attention to uncover the molecular mechanisms underpinning cytochrome *c* release and activation. The fact that MOMP is not always complete, and does not always lead to cell death, suggests that the response to MOMP is nuanced and opens questions regarding mitochondrial dynamics and metabolism,^123^ the impact of partial MOMP on the cell, and how it can be exploited for clinical translation.
